# 3D Printable Poly(*N*-isopropylacrylamide) Microgel Suspensions with Temperature-Dependent Rheological Responses

**DOI:** 10.1021/acsapm.3c03230

**Published:** 2024-03-21

**Authors:** Zhecun Guan, Sai Krishna Katla, Vidumin Dahanayake, Jinhye Bae

**Affiliations:** † Department of NanoEngineering, University of California San Diego, La Jolla, California 92093, United States; ‡ Anton Paar USA, Inc., Ashland, Virginia 23005, United States; § Chemical Engineering Program, University of California San Diego, La Jolla, California 92093, United States; ∥ Materials Science and Engineering Program, University of California San Diego, La Jolla, California 92093, United States

**Keywords:** Microgels, stimuli-responsive hydrogels, rheological response, temperature responsiveness, 3D printing

## Abstract

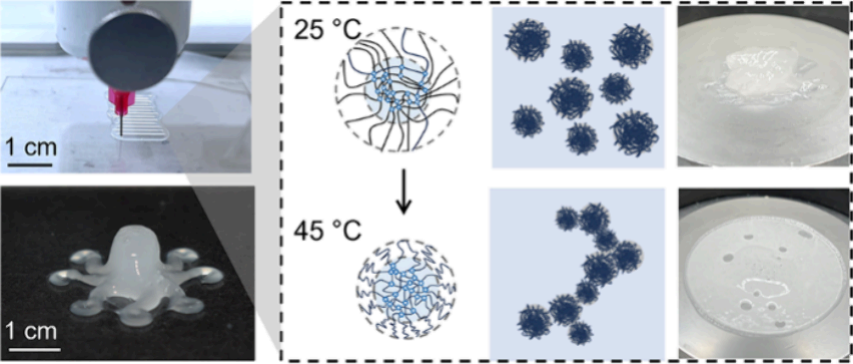

Microgel suspensions have garnered significant interest in fundamental research due to their phase transition between liquid-like to paste-like behaviors stemming from tunable interparticle and particle–solvent interactions. Particularly, stimuli-responsive microgels undergo faster volume changes in response to external stimuli in comparison to their bulk counterparts, while maintaining their structural integrity. Here, concentrated and diluted suspensions of poly(*N*-isopropylacrylamide) (PNIPAm) microgels are dispersed to different packing fractions in water for the characterizations of temperature-responsive rheological responses. In the intrinsic volume phase transition (VPT), polymer chains collapse, and microgels shrink to smaller sizes. Additionally, the intermicrogel and microgel–solvent interactions vary in VPT, which results in microgel clusters that significantly affect the linear shear moduli of suspensions. The effect of the temperature ramp rate of PNIPAm microgel suspensions on rheological responses is characterized. Moreover, the effect of the mass fraction of microgels on the relative viscosity of dilute microgel suspensions is investigated. These results shed light on understanding the heating and cooling rate-dependent temperature responsiveness of PNIPAm microgel suspensions, establishing pathways to regulate the rheological characteristics in temperature-responsive microgel-based platforms. Therefore, this work envisions technological advancements in different fields such as drug delivery, tissue engineering, and diagnostic tools.

## 1 Introduction

Hydrogels are polymeric materials that retain water and thus exhibit softness, deformability, and biocompatibility^1−3^ and typically form nanoscale porosity inside of their mesh-like matrix.^4^ Bulk hydrogels are not suited for their intended application scenarios when miniaturization and rapid mass transport are required. Due to the interstitial dense structure and nanometer scale mesh size, the mass transport rate in bulk hydrogels is typically limited when responding to environmental changes, such as temperature, light, humidity, pressure, electrical field, and magnetic field.^5,6^ On the other hand, microgels, also known as hydrogel microparticles, demonstrate similar water retainment with bulk hydrogels but exhibit miniaturized size of ∼1–1000 μm.^7,8^ Such large interfacial area and microscale porosity due to the void space that inherently exists between microgels after packing enable faster mass transfer in microgels compared with bulk hydrogel.^9,10^ Stimuli-responsive microgels thus present fast responses in the vicinity of various stimuli, which embodies the toolbox for the on-demand realization of myriad applications.^11^

Poly(*N*-isopropylacrylamide) (PNIPAm) has been extensively studied for its temperature responsiveness due to the lower critical solution temperature (LCST). At temperatures below the LCST, the polymer retains hydration while transitioning to a relatively hydrophobic state above this threshold. This transition occurs due to the disruption of hydrogen bonds, which leads to changes in solubility and chain conformation.^12−14^ In a similar manner, cross-linked PNIPAm encounters volume phase transition (VPT) and transforms from a swelled to a collapsed state when the temperature is higher than the volume phase transition temperature (VPTT).^13^ Pelton and Chibante reported the preparation of monodispersed PNIPAm nanogels with an average diameter of 500 nm by surfactant-free precipitation polymerization which is conducted above the LCST, commonly at 70 °C to exploit the globule-to-coil transition of polymer chains.^15,16^ Using a similar preparation method, Senff and Richtering observed a decrease in the hydrodynamic radius of PNIPAm nanogels with increasing temperature and obtained a fitted relation between the volume fraction and the viscosity of nanogel suspensions.^17^ In another work, they examined the effect of cross-linking density on the rheological behaviors of PNIPAm nanogel suspensions.^18^ The repulsive interparticle interaction and van der Waals attraction in PNIPAm nanogel suspensions are then discussed as a function of temperature.^19,20^ PNIPAm and PNIPAm-based nanogels have been particularly attractive in cargo systems, such as artery embolization and drug delivery, because of their volume change-induced uptake and release upon temperature responsiveness.^21,22^ However, PNIPAm nanogels exhibit limitations in biomedical fields such as encapsulating cells and favoring cell proliferation as scaffolds as the nanogel size is much smaller than cells.^23,24^

In recent decades, PNIPAm microgels have been synthesized and investigated. Dowding and co-workers prepared PNIPAm microgels with an average diameter of 2.5 μm using inverse suspension polymerization.^25^ Márquez and co-workers synthesized PNIPAm microgels with a diameter range of 8–60 μm and studied the swelling kinetics of microgels.^26^ They derived a network diffusion coefficient of PNIPAm microgels by analyzing the swelling and deswelling process from the recorded video using optical microscopy. Unlike nanogel suspensions that can undergo crystallization with high particle volume fractions above around 0.5 due to their monodispersity,^27^ gravitational forces predominate over the thermal fluctuations when the diameter of microgels is greater than 10 μm, which allows for particle settling.^28^ To the best of our knowledge, rheological responses of polydisperse PNIPAm microgel suspensions have not been well studied. Here, we prepared concentrated and diluted PNIPAm microgel suspensions of different cross-linking densities to gain a comprehensive understanding of temperature-induced size, clustering, and rheological behavior changes in temperature-responsive microgel suspensions. Temperature significantly influences the oscillatory sweep and viscosity of microgel suspensions by tuning the microgel sizes. Taking advantage of this temperature responsiveness, 3D printability of concentrated microgel suspension inks is demonstrated. Additionally, the temperature ramping rate, which can be correlated to water diffusion kinetics in microgels, has been further proven to impact linear shear moduli of PNIPAm microgel suspensions over multiple heating and cooling cycles. Lastly, a linear relationship between relative viscosity and mass fraction of PNIPAm microgels is observed in diluted microgel suspensions. Our study enhances the understanding of thermosensitive microgel suspension platforms, offering potential utility across a broad range of applications in biomedical engineering, soft actuators, and wearable sensors.

## 2 Materials and Methods

### 2.1 Materials

*N*-Isopropylacrylamide monomer (NIPAm, 98.0%, M_W_ = 113.16 g mol^–1^ stabilized with 4-methoxyphenol (MEHQ)) was purchased from Tokyo Chemical Industry (TCI) America. *N*,*N*-Methylenebis(acrylamide) (BIS, 99.5%), 2-hydroxy-4′-(2-hydroxyethoxy)-2-methylpropiophenone (Irgacure 2959, I-2959, 98%), and Span 80 were purchased from Sigma-Aldrich. Mineral oil (light) was obtained from Thermo Fisher Scientific. All chemicals were used as received without further purification.

### 2.2 Preparation of PNIPAm Microgels and Microgel Suspensions

PNIPAm microgels were synthesized from the water-in-oil template reported in our previous work.^29^ The aqueous phase, PNIPAm precursor solution, was prepared by mixing a monomer (NIPAm, 2 M), a cross-linker (BIS, 0.13 M) and a photoinitiator (I-2959, 0.4 wt %) following the compositions shown in Table 1 in deionized (DI) water using a vortex mixer (MX-S, Dlab, USA) until all chemicals dissolved at 23 °C. The prepared microgels are named by the molar ratio between the monomer and the cross-linker (i.e., MG640 and MG320). The oil phase containing 10 mL of mineral oil and surfactants (Span 80, 200 μL) was transferred to a 15 mL centrifuge tube (SPL Life Science Co., Ltd.), mixed using a vortex mixer, and purged with nitrogen for 10 min. Then, 1 mL of PNIPAm precursor solution and 4 mL of oil phase were slowly added into a glass Petri dish and magnetically stirred at 100 rpm for 5 min. The mixed emulsion was irradiated with UV light (Omnicure S2000, wavelength: 365 nm, Lumen Dynamics, Canada) at 300 mW cm^–2^ for 15 min. After polymerization, the cross-linked PNIPAm microgels were collected in a 15 mL centrifuge tube and rinsed three times with 10 mL of mineral oil to remove the surfactants by vortex mixing for 1 min and centrifugation at 2500 rpm for 5 min. After that, the microgel suspensions were washed using DI water three times to fully remove the unreacted monomer by vortex mixing for 1 min and centrifugation at 5000 rpm for 5 min. Fourier-transform infrared spectroscopy (FTIR, PerkinElmer Inc., USA) was conducted to make sure all impurities were removed. All the measurements were performed at 23 °C unless otherwise noted.

**Table 1:** Quantities of Reagents Used in Preparation of PNIPAm Microgels and Layers

Code	NIPAm solution, (2 M)/mL	BIS solution, (0.13 M)/μL	I-2959/g	Molar ratio of NIPAm: BIS	Linear swelling ratio, *λ*_*S*_	Linear deswelling ratio, *λ*_*D*_
MG640	5	120	0.02	640	1.419 ± 0.013	0.716 ± 0.019
MG320	5	240	0.02	320	1.306 ± 0.010	0.778 ± 0.008

Mass fraction is used to quantify the mass of microgels in microgel suspensions as the microgel interpenetration should be considered in soft colloidal systems, especially for microgels of low cross-link densities.^30^ To determine the weight of dried microgels, the fully swollen microgels were frozen using liquid nitrogen and placed in a freeze-dryer (FreeZone 2.5, Labconco, USA) at 0.03 mbar and −80 °C for at least 5 days to fully dry the microgels. A white powder was obtained and thereafter protected from humidity until its utilization. For concentrated suspensions, MG640 and MG320 samples were dispersed in DI water and centrifuged at 7500 rpm for 10 min to achieve a concentrated state. After removing the supernatant, concentrated microgel suspensions were collected and stored at 4 °C for future use. The mass fraction of concentrated microgel suspensions was calculated at 4.48 ± 0.15 wt % from three batches. The freeze-dried microgels were diluted to various mass fractions from 0.05 to 1 wt % to formulate dilute microgel suspensions.

### 2.3 Size Distribution of Prepared PNIPAm Microgels and Microgel Clusters

Optical microscopy (Eclipse Ni, Nikon, Japan) was used to characterize the size distribution of fully swelled MG640 and MG320 microgels. The images were analyzed through NIS-Elements software, and over 100 microgels for each data set were measured. Temperature controller (TC-1-100s, Bioscience Tools, USA) was employed to accurately control the ambient temperature of microgel suspensions while imaging. To clearly show the margin of microgels, a threshold plug-in in ImageJ was employed to process the optical micrographs. The coefficient of variation (CV) is used to quantify the polydispersity of fabricated PNIPAm microgels, where σ is the standard deviation, and μ represents the mean.
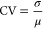


In addition, a dynamic image analyzer (Litesizer DIA 500, Anton Paar GmbH, Austria) was employed to investigate the average hydrodynamic diameter of the microgel clusters via the analysis of their direct images. A liquid flow dispersion unit containing 600 mL of DI water fully dispersed the concentrated microgel suspensions of 1–2 mL to reach a volume ratio of ∼0.2 vol %. The temperature of the microgel solution was controlled using an external heater with feedback from a thermocouple. Using an additional heater and the temperature feedback loop, it was difficult to adjust the microgel suspension to expected temperatures; therefore, the measurements for MG640 and MG320 samples were not at the exact same temperatures. The high-speed camera captured the microgel clusters while being circulated within the instrument, and these dynamic images were analyzed for the hydrodynamic diameters of microgel clusters and were recorded using Kalliope Software from Anton Paar. Each data point contains three measurements.

### 2.4 Preparation of Casted PNIPAm Layers

We used a sandwich molding method to cast PNIPAm layers. PNIPAm precursors were prepared using the same recipes shown in Table 1 as PNIPAm microgel synthesis. The precursors were injected in between two glass slides, with the other two glass slides functioning as a spacer of 1 mm. The width and length of the as-prepared PNIPAm layer are 1 and 2 cm, respectively. Molded precursors were polymerized and cross-linked with a UV intensity of 100 mW cm^–2^ for 20 min. After that, the layers were transferred to Petri dishes filled with DI water, and each PNIPAm layer was biopsied to three spherical samples with a diameter of 0.5 cm. The samples were swelled overnight at 23 °C and then heated to 50 °C for 5 h to finish isotropic-free swelling and deswelling. Linear swelling and linear deswelling ratios were calculated based on the measured lateral length via a caliper. Young’s moduli of PNIPAm layers at swelled and deswelled states were measured using a nanoindenter (Optics11, Piuma, USA) with probe stiffnesses at 0.014 and 0.028 N m^–1^.

### 2.5 Rheological Measurements of Microgel Suspensions

Rheological data were obtained from an ARG2 rheometer by TA Instruments with a plate–plate parallel geometry (aluminum, 40 mm diameter) and a gap of 600 μm. The solvent trap of the geometry is filled with DI water during the measurement to prevent water evaporation by an immersion ring and a chamber around the Peltier plate of the rheometer. Microgel suspension samples were first transferred onto the bottom plate of the rheometer using a spatula and then compressed by lowering the geometry. To determine the linear viscoelastic region (LVR), we conducted amplitude sweeps with oscillation strains covering a range from 0.01% to 1000% at 10 rad/s. Linear shear moduli of concentrated MG640 and MG320 PNIPAm microgel suspensions, including storage moduli (*G*′) and loss moduli (*G*′′), are then measured by oscillatory frequency sweeps at 25 and 45 °C, respectively, with frequencies from 0.001 to 100 Hz at a low strain amplitude of 1% within LVR. After that, an oscillation temperature ramp was performed at a ramp rate of 1, 3, and 5 °C/min from 25 to 45 °C for four complete cycles at a shear rate of 10 rad/s and a strain of 1%. Frequency sweeps and amplitude sweeps were conducted using the same batches of microgel suspensions for consistency. To prove the accuracy of Peltier temperature control in the rheometer at a high heating rate of 5 °C/min, the temperature sequence from 25 to 45 °C was recorded by a thermometer when heating using the rheometer.

### 2.6 Extrusion-Based 3D Printing

Concentrated PNIPAm microgel suspensions were printed by extrusion using a 3D printer (BioX, Cellink, USA). MG320 suspensions were transferred to the 3 mL syringes and centrifuged at 2500 rpm for 3 min to remove the air bubbles from the ink. The ink was placed in the extrusion carriage of the 3D printer and printed on a glass slide using a 27-gauge needle (27G, inner diameter of 0.21 mm) and a printing bed temperature at 23 °C at a 10 mm s^–1^ deposition rate with a pneumatic pressure of 50 kPa for printability assessment at different syringe temperatures. The needle was then changed to 25G (inner diameter of 0.26 mm) to avoid the potential clogging in printing more complex structures and various temperature profiles including changes in both syringe temperature and printing bed temperature.

### 2.7 Differential Scanning Calorimetry (DSC) Measurements

The VPTT measurements of PNIPAm microgel suspensions were carried out on a DSC (Pyris Diamond DSC, USA) under a nitrogen atmosphere at different ramp rates of 1, 3, and 5 °C/min from 10 to 50 °C, each containing two heating and cooling cycles. Microgel suspensions were sealed in the aluminum container to avoid water evaporation during the measurement. The baselines at each ramp rate were measured and deducted from the results using an empty reference sample before the thermographs of microgel suspension were measured. The flow rate of N_2_ was adjusted to 20 mL/min; the purity of N_2_ ≥ 99.999%.

### 2.8 Contact Angle Measurements

The contact angles of PNIPAm layers are measured by Goniometer (Model 200, ramé-hart instrument, USA). Here, a 15 μL DI water droplet was dropped on a casted PNIPAm layer surface that shares the same recipe with MG640 or MG320 using a microsyringe (Gilmont). The real-time temperature of PNIPAm layers is recorded by a laser infrared temperature gun (Lasergrip 1080, USA). The reported values for contact angles are averaged over at least three independent measurements.

### 2.9 Statistical Analysis

Data are presented as mean ± standard deviation (SD). A minimum of three tests was performed for all experiments to ensure that the results reported were significant.

## 3 Results and Discussion

### 3.1 Fabrication and Characterization of PNIPAm Microgel Suspension

PNIPAm colloids have gained significant attention and thus have been intensively examined due to their temperature responsiveness. To date, a wide size range from nanometer to millimeter scale of PNIPAm colloids has been prepared using multiple fabrication methods. The hydrophilic-to-hydrophobic transition of PNIPAm polymer chains allows for surfactant-free precipitation polymerization of the monodispersed PNIPAm nanogels in an aqueous phase above LCST.^21^ In the surfactant-mediated fabrication, the role of surfactant as well as subsequent phase transition of PNIPAm nanogels has been reported. The introduction of anionic surfactants, such as sodium dodecyl sulfate, promotes the swelling of the nanogels and shifts the transition to a higher temperature. On the other hand, cationic surfactant, such as dodecylpyridine bromide, impacts less on the swelling and phase transition because of the electron-rich amide group in PNIPAm.^31^ However, the prepared colloids are usually limited to the nanoscale owing to the high polymerization temperature-induced hydrophobicity of polymer chains above LCST.

**Figure 1:**
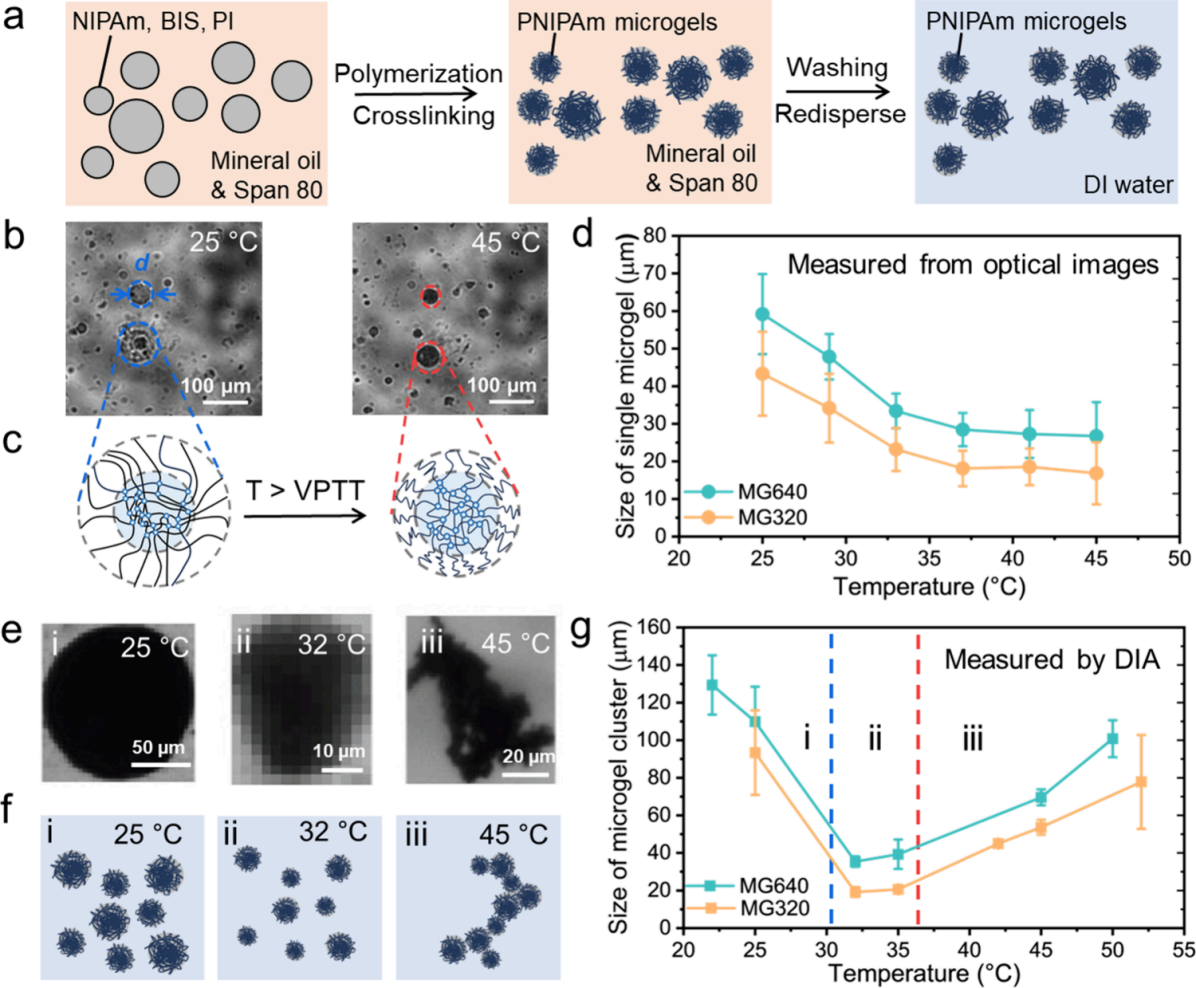
Fabrication of PNIPAm microgel suspensions and their temperature responsiveness. (a) Schematic of the preparation of PNIPAm microgel suspensions using a water-in-oil template. PNIPAm precursor droplets are polymerized and cross-linked upon UV irradiation to form microgels. PNIPAm microgel suspensions are obtained by washing and redispersing microgels to DI water with different mass fractions. (b) Micrographs of MG640 microgels in diluted suspensions at 25 and 45 °C, respectively. Diameters (*d*) of microgels are measured from optical images using ImageJ. (c) Corresponding schematics of the size change of PNIPAm microgels upon VPTT. (d) Effect of temperature on the size of the single microgel in diluted MG640 and MG320 suspensions. (e) Representative images of the microgel clusters in water recorded with a high-speed camera using DIA at (i) 25 °C, (ii) 32 °C, and (iii) 45 °C. (f) Corresponding schematics of microgel clustering at (i) 25 °C, (ii) 32 °C, and (iii) 45 °C. (g) Effect of temperature on the size of microgel clusters in diluted MG640 and MG320 suspensions. The average hydrodynamic diameters of the microgel cluster are measured from DIA using Anton Paar’s Kalliope Software.

To obtain a microscale system, we select a room-temperature batch emulsification method to prepare PNIPAm microgels via a water-in-oil template. At room temperature, microgel size is mainly determined by the size of the water-in-oil droplet, which can be adjusted by the amount of surfactant and emulsification method, instead of the hydrophilic-to-hydrophobic transition of PNIPAm chains.^32^ The aqueous PNIPAm precursor is composed of *N*-isopropylacrylamide (NIPAm) as a monomer, *N*,*N*-methylenebis(acrylamide) (BIS) as a cross-linker, and 2-hydroxy-4′-(2-hydroxyethoxy)-2-methylpropiophenone (Irgacure 2959, I-2959) as a photoinitiator. Mineral oil is used as the oil phase because it has a much lower O_2_ solubility (i.e., 0.134 ± 0.004 at 24 °C^33^) than other types of oil commonly used in emulsification methods, which ensures that radical polymerization reactions are not inhibited by the presence of oxygen.^34,35^ Water-in-oil emulsions are prepared by magnetically stirring the mixture of the aqueous PNIPAm microgel precursor solution and the oil phase containing nonionic Span 80 surfactants (Figure 1a). Upon UV irradiation, the precursor droplets are photopolymerized and cross-linked to PNIPAm microgels. Based on the molar ratio of the monomer and the cross-linker in the aqueous phase, prepared PNIPAm microgels are noted as MG640 and MG320 (Table 1). MG640 refers to a higher monomer to cross-linker ratio and thus a lower cross-linking density than MG320. These fabricated microgels are washed using mineral oil and DI water three times each before freeze-drying. The freeze-dried microgels are then diluted to different mass fractions for diluted microgel suspensions. For concentrated microgel suspensions, we employ fully swelled PNIPAm microgels after water washing and collect the microgel suspensions at the bottom of the centrifuge tube after centrifugation at 7500 rpm for 10 min. The complete removal of the redundant surfactants and polymerization of the PNIPAm precursor is confirmed by FTIR (Figure S1). Both C–H stretching and C–O stretching vibrations of Span 80 with characteristic peaks at 2855 and 1173 cm^–1^ disappear, respectively, after washing and freeze-drying.^36^ Additionally, peaks in the range of 800–1000 cm^–1^ characterizing the stretching mode of vinyl double bonds disappeared, indicating that polymerization and cross-linking have taken place.^37^

To observe the size information on the single microgel, DI water is added to each batch of concentrated microgel suspensions to dilute them to a 20:1 ratio and allow microgels to fully swell for 24 h at 23 °C before size measurements. The size distribution of PNIPAm microgels at their equilibrium state was characterized by optical microscopy, and over 100 microgels were measured via ImageJ. Using a temperature controller, microgel suspension can be photographed *in situ*. The diameter of the circled MG640 microgels decreases from 52 to 36 μm and 79 to 55 μm, respectively, when locally heated from 25 to 45 °C (Figure 1b). Analogous to PNIPAm nanogels, microgels exhibit a decreasing cross-linking density radially with the distance from the center of the microgel as cross-linker is consumed more rapidly than NIPAm monomer.^38,39^ Therefore, the dehydration of heterogeneous “core–shell” structure PNIPAm microgels can be depicted as the shrinkage of the linear polymer chains that dangle from the densely cross-linked core (Figure 1c).^40^ The average diameters of MG640 and MG320 are recorded as a function of temperature with an interval of 4 °C (Figure 1d). Contrary to bulk hydrogels that perform a sharp transition at the LCST, temperature elevation results in a gradual decrease in the diameters of MG640 microgel, from 59 ± 11 to 27 ± 9 μm, exhibiting a continuous phase transition possibly because of the irregular microgel surface and microgel nonuniformity.^10^ The polydispersity of MG640 and MG320 microgel sizes at different temperatures is quantified by the coefficient of variation (CV) in Figures S2 and S3. The CV of MG640 microgels at 25 °C is 17.96%, while the results for relatively smaller microgels are 23.39% at 41 °C and 33.74% at 45 °C. Similarly, the CV values for MG320 microgels are 20.42% at 25 °C and 29.22% at 45 °C.

Beyond VPTT, PNIPAm microgels tend to dehydrate, and the increasing hydrophobicity of polymer chains leads to the formation of microgel clusters. To visualize the real-time formation of microgel clusters in water, a dynamic image analyzer (DIA) is used to record the morphology and the average size of microgel clusters using a high-speed camera. The as-prepared microgel suspension is diluted to ∼0.2 vol % in water to optimize the concentration for capturing photographs using the DIA instrument. An external thermal probe and heater are used on the sample tank of the DIA to control the temperature. As DIA requires water flow to acquire particle images, and microgel clusters may be disturbed, resulting in smaller observed sizes compared to conditions without turbulence. Figure 1e shows the representative images of MG640 microgel clusters taken at 25, 32, and 45 °C, which is also schematically depicted in Figure 1f. At 25 °C, microgels are well dispersed in water in their swelled state. When the temperature is increased to their VPTT, microgels collapse and shrink in volume but remain dispersed, while they lose the affinity to water and form microgel clusters with other microgels as temperature increases. The size of microgel clusters at 32 and 45 °C can be approximately fitted with the Gaussian function, while performing a high polydispersity at 25 °C (Figures S4 and S5). To confirm the transition to a microgel cluster macroscopically, a low-magnification video is taken for the cooling process in the air from 45 to 25 °C in microgel suspensions (Video S1). It is difficult to differentiate microgels from water at 25 °C under transmittance light, while the appearance and disappearance of microgel clusters are explicitly recorded. The suspension also turns from translucent to transparent upon cooling. Elevated temperatures result in microgel shrinkage due to network collapse with the progressively decreasing attractive strengths between the amine groups and environment water.^41^ This gives rise to attractive interaction among the hydrophobic isopropyl groups within microgels which leads to particle aggregations. Figure 1g summarizes three measurements at each temperature condition for MG640 and MG320 microgels. Based on the interparticle attraction, the temperature range is categorized into three regions: (i) repulsive force-dominated area, (ii) near VPTT, and (iii) attraction force-dominated area. In region (i), the hydrogen bonding between microgels and water facilitates microgels to disperse. Region (ii) is the proximity of VPTT where the repulsive and attractive strengths reach a balance, where the microgel shrinks but remains well dispersed in water. However, when the temperature is above VPTT, microgels aggregate and form clusters but are disturbed by the water flow when imaging, leading to the size increase in region (iii) while still smaller than region (i).

### 3.2 Temperature-Dependent Rheological Responses of Concentrated PNIPAm Microgel Suspensions

**Figure 2:**
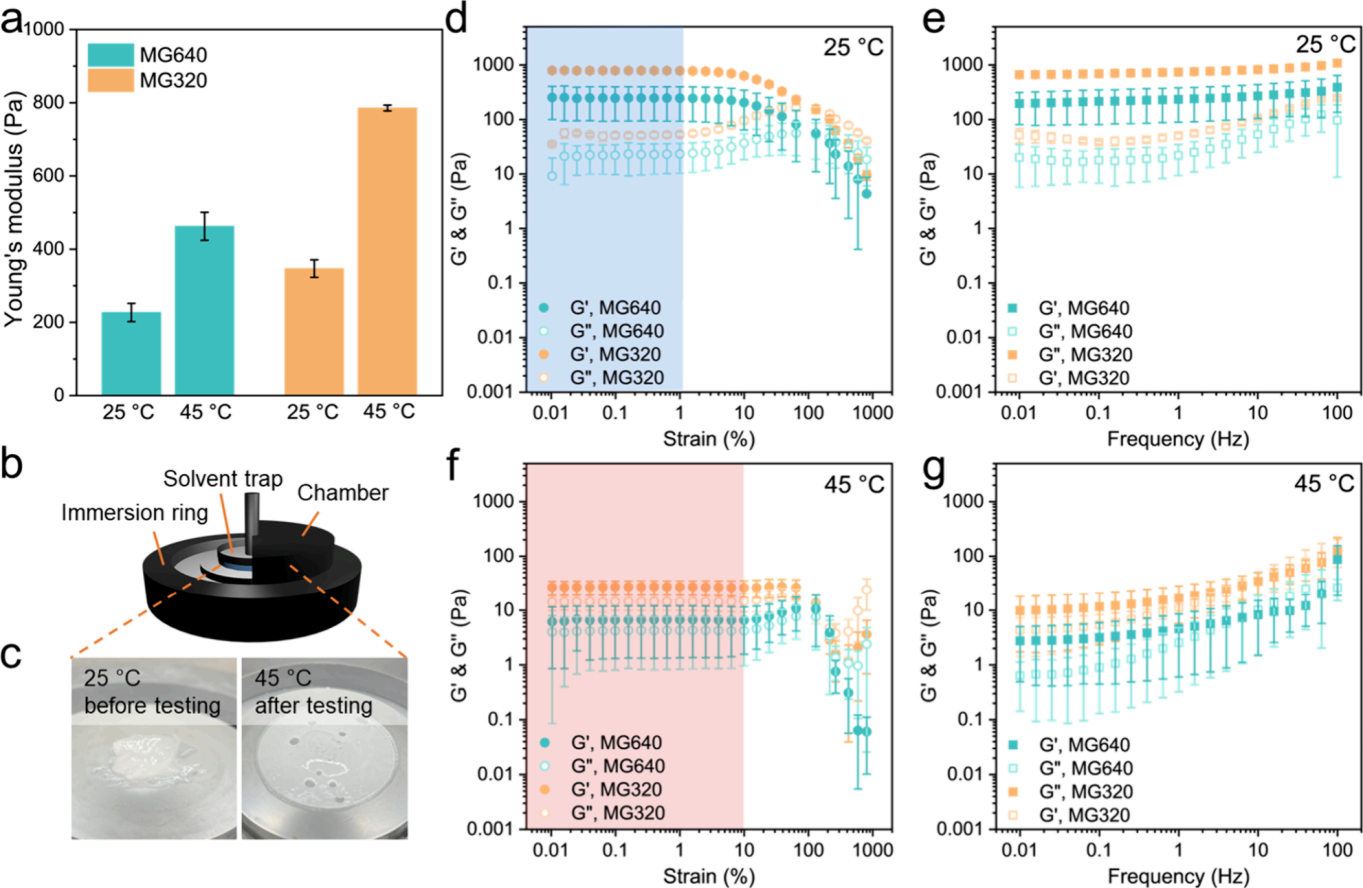
Effect of temperature on rheological behaviors of concentrated PNIPAm microgel suspensions with different cross-link densities. (a) Young’s moduli of PNIPAm layers prepared from MG640 and MG320 recipes at 25 and 45 °C, respectively. (b) Schematic of the evaporation-avoid rheometer setup. (c) Photographs of concentrated MG640 suspensions before (25 °C) and after testing (45 °C). (d) Oscillation amplitude of concentrated MG640 and MG320 suspensions that determine *G*′ (solid circles) and *G*′′ (hollow circles) at 25 °C. The blue shade area indicates LVR at 25 °C. (e) Oscillatory frequency sweep of concentrated MG640 and MG320 suspensions at 25 °C. (f) Oscillation amplitude of concentrated MG640 and MG320 suspensions at 45 °C. The red shade area indicates LVR at 45 °C. (g) Oscillatory frequency sweep of concentrated MG640 and MG320 suspensions at 45 °C.

With a lower cross-linking density, MG640 microgels exhibit lower Young’s moduli and higher deformability than MG320. Young’s modulus of a single microgel is technically difficult to measure; thus, casted PNIPAm layers that share the same recipes with MG640 and MG320 microgels have been fabricated. Each PNIPAm layer is prepared with a thickness of 1 mm, a length of 2 cm, and a width of 1 cm. Linear swelling and deswelling ratios quantify the lateral swelling and deswelling of the PNIPAm layers. Linear swelling ratio (*λ*_*S*_) is defined as the ratio of the lateral length at the swelled state to that of the as-prepared state, and the linear deswelling ratio (*λ*_*D*_) is defined as the length of the deswelled state to that of the as-prepared state. The *λ*_*S*_ values for MG640 and MG320 PNIPAm layer are measured after overnight swelling in water at 23 °C, and *λ*_*D*_ values are recorded after deswelling overnight at 45 °C to achieve the equilibrium dimension (Table 1). At the fully swelled state, the PNIPAm layer prepared from MG640 recipe demonstrates an average Young’s modulus of 227 Pa, and this value significantly increases to 462 Pa at 45 °C. Similarly, the MG320 layer presents 347 and 786 Pa at 25 and 45 °C, respectively (Figure 2a).

When the diameter of the microgel is larger than 10 μm, van der Waals attraction forces are negligible (≪*k*_B_*T*) when compared with friction between adjacent microgels.^42−44^ Therefore, microgels can be concentrated and form a jammed state when the packed microgels are immobilized by their surrounding microgels through physical interactions and transfer from a liquid-like state to a solid-like paste when the volume fraction of microgels is higher than 0.58,^45^ which is defined as random loose packing. This characteristic of concentrated microgel suspensions has recently been exploited for ink materials in three-dimensional (3D) printing.^7^ Specifically, the PNIPAm microgel surface is associated with negative charges as a result of the initiator used, which prevents them from aggregation.^39^ Jamming of microgels can thus be simply achieved by centrifugation. At packing densities over 0.58, microgel suspensions exhibit viscoelastic properties, displaying pronounced temperature dependence. Consequently, rheological measurements are performed to elucidate the temperature-induced microgel–microgel and microgel–solvent interactions in the concentrated PNIPAm microgel suspensions. A plate–plate parallel geometry (aluminum, 40 mm diameter) with a gap of 600 μm is harnessed for all rheological measurements. Note that the solvent trap of the geometry is filled with DI water to prevent suspensions from continuous evaporation upon heating by an immersion ring and a chamber around the Peltier plate of the rheometer (Figure 2b).^46^ With this setup, a concentrated PNIPAm microgel suspension is photographed at 25 °C, before oscillation amplitude and sweep tests, and at 45 °C after tests, respectively (Figure 2c). The concentrated suspension turns opaque uniformly after heating to 45 °C for 20 min.

To determine the LVR, concentrated MG640 and MG320 suspensions are transferred to the closed chamber and measured at an oscillation strain covering 0.01% to 1000%. At 25 °C, the shear elastic modulus (*G*′) and viscous modulus (*G*′′) of the suspension perform a plateau ranging from 0.01% to 1% on a logarithmic scale, corresponding to the LVR in which *G*′ overwhelms *G*′′ and *G*′ reaches 252 Pa for MG640 suspensions (Figure 2d). Similar LVRs were observed in concentrated MG320 suspensions, therefore all oscillatory frequency sweeps were performed at a low strain amplitude of 1%, spanning frequencies of 0.01 to 100 Hz (Figure 2e). At small amplitude oscillatory frequency sweeps (0.01–10 Hz), *G*′ is one magnitude larger than *G*′′, and they both remain at a similar value which corresponds to plateau modulus (*G*_*p*_). When the frequency is raised higher than 10 Hz, there is an increase in both *G*′ and *G*′′. Concentrated MG640 suspension exhibits LVR over 0.01% to 10% strain, and *G*′ remains at around 6 Pa in this range at 45 °C (Figure 2f). Notably, the difference between *G*′ and *G*′′ of the MG640 suspension significantly decreases from 243 to 2 Pa from 25 to 45 °C, indicating a solid-like paste to liquid-like transition in suspensions. In Figure 2g, *G_p_* of concentrated MG320 suspension decreases from 202 to 3 Pa from 25 to 45 °C. *G*′′ exceeds *G*′ starting from 10 Hz in concentrated MG640 and MG320 suspensions, which results in viscoelastic liquid behaviors.

**Figure 3:**
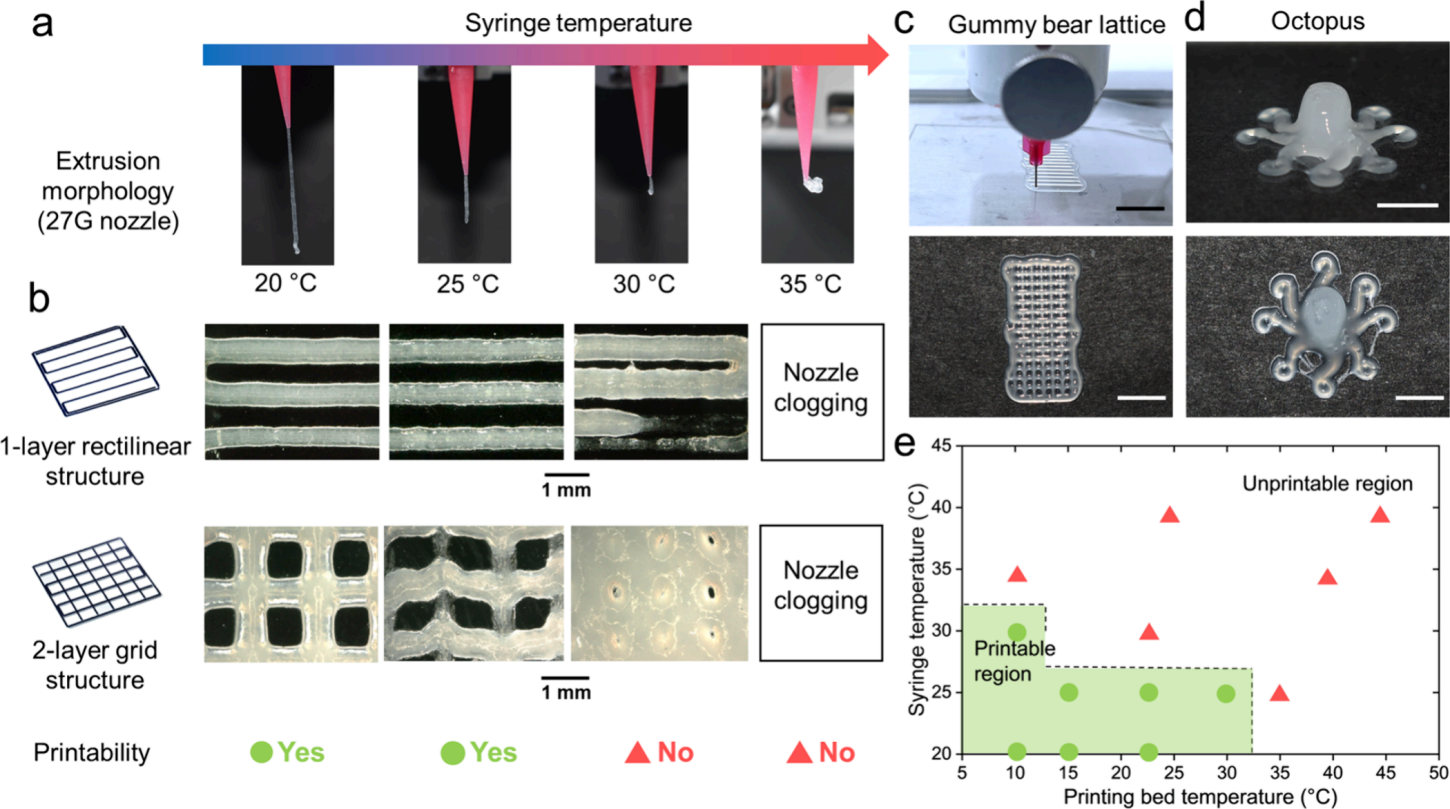
3D printing of concentrated PNIPAm MG320 suspensions. (a) Extrusion morphology of the MG320 ink using a 27G nozzle. (b) Printing results of 1-layer rectilinear structure and 2-layer grid structure using MG320 ink at different syringe temperatures. (b) Photographs of the printing process and the top view of the printed lattice structure of the gummy bear. Scale bar = 1 cm. (c) Side view and top view of the 3D printed octopus structure. Scale bar = 1 cm. (d) Printability diagram of MG320 ink as a function of the syringe and printing bed temperatures. The dashed lines indicate a boundary between printable inks with unprintable inks.

After confirming the temperature-responsive rheological responses of concentrated PNIPAm microgel suspensions, we further investigated this temperature effect on the printability of inks in extrusion-based 3D printing. Printability is defined as the ability to extrude from a nozzle where the solid-like behavior (*G*′ > *G*′′) of the ink is required to retain the shape of the extruded material, therefore closely related to the printing temperature.^47^ Two temperature parameters, syringe temperature and printing bed temperature, are studied in the 3D printing of this temperature-responsive ink. The syringe temperature is controlled by the printing carriage holding the syringe. This temperature determines the ink temperature upon extrusion from the nozzle, while the printing bed temperature remains at room temperature. The extrusion morphology of concentrated MG320 suspensions using a 27G nozzle was recorded at different syringe temperatures (Figure 3a). The extrusion is continuous and fluent at 20 and 25 °C, while becoming discontinuous and nonuniform at higher temperatures. Here, 1-layer rectilinear and 2-layer grid paths were printed, respectively, to assess the printability of the ink. The printing follows the traces with good printability at 20 and 25 °C (Figure 3b). A 3-layer grid structure was also printed (Video S2) with a layer-by-layer printing path. When the syringe temperature reaches 30 °C, the ink performs discontinuity in printing the rectilinear shape and poor shape fidelity in the printing grid structure, which are categorized as an unprintable condition. Fixing the syringe temperature at 20 °C, more complex structures including gummy bear-shaped lattice and 3D octopus were designed and printed (Figure 3c, d). The clear printing paths of these structures confirm the excellent 3D printability and shape retention of our inks, especially from the side views. We then performed a similar printing assessment as in Figure 3a to determine the effect of printing bed temperature on printability. The printing bed functions to support the inks after extrusion until the completion of printing; therefore, the printing bed temperature impacts the postextrusion stability and layer stacking. In Figure 3e, the printability diagram summarizes the printability of concentrated MG320 suspensions as a function of the syringe and printing bed temperatures. The temperature profiles are categorized as printable and unprintable types, and both temperatures below VPTT of PNIPAm microgels are required to facilitate printing.

To investigate the microgel–solvent interaction in microgel suspensions, contact angle measurements are performed to characterize the wettability of water on PNIPAm layers as a function of temperature. The temperature of the PNIPAm layer is measured using a laser infrared temperature gun. The contact angle of water on the MG640 PNIPAm layer at 21.1 °C is 25.4 ± 1.4°, while this value is 92.3 ± 0.7° at 44.8 °C (Figure 3a). A similar trend is observed in the MG320 PNIPAm layer with contact angles of 32.0 ± 2.3° at 22.2 °C and 97.9 ± 1.1° at 45.0 °C. Water droplets show a relatively greater wettability at lower temperatures while the PNIPAm–water interaction becomes repulsive beyond VPTT. In addition, MG320 layers perform a higher hydrophobicity compared with MG640 PNIPAm layers upon heating, which also confirms the assumption that interaction between water and PNIPAm microgels impacts the microgel sizes and the formation of microgel clusters.

Typically, PNIPAm bulk structures swell and deswell below and above 32 °C, respectively.^13^ The colloidal PNIPAm counterparts, however, do not present such a sharp transition despite identical chemical composition and qualitatively similar swelling behaviors.^48,49^ Suárez and co-workers have derived a network diffusion coefficient for swelling kinetics of PNIPAm microgels of 1.0 × 10^–10^ m^2^ s^–1^,^26^ which is in between the water self-diffusion coefficient (2.3 × 10^–9^ m^2^ s^–1^ at 25 °C) and the value for bulk PNIPAm (5.0 × 10^–11^ m^2^ s^–1^ at 25 °C).^50,51^ The equilibrium swelling and deswelling time are therefore about 1 s for PNIPAm microgels. In general, PNIPAm-based hydrogels exhibit thermal hysteresis in temperature ramps, and the temperature difference (Δ*T*) for PNIPAm hydrogels before and after the VPT during heating and cooling is about 1–2 °C, which is generated from the delayed dissolution of the polymer chains in a shrunken state.^52^

Nonetheless, the repeatable temperature responsiveness of concentrated microgel suspensions has been scarcely investigated as the amount of water is limited compared with their dilute counterparts. Therefore, the kinetics and repeatability of the swelling and deswelling of microgels in concentrated microgel suspensions are explored for a better understanding of the temperature-responsive rheological behaviors. DSC analysis was performed for two cycles to characterize the VPTT of concentrated PNIPAm microgel suspensions. Sealed in aluminum containers, PNIPAm microgel suspensions experience two cycles of heating and cooling between 10 and 50 °C at a ramp rate of 1, 3, and 5 °C/min. A peak of an exothermic nature is observed in correspondence with the VPTT, noted as *T*_exo_, indicating the transition from the swollen to the shrunken state of microgels during heating. Microgels absorb energy to reach a new thermoreversible state characterized by reduced size, changed mutual interactions, and spatial configurations. Similarly, the VPTT in the endothermic process is noted as *T*_endo_. In the MG640 suspension, *T*_exo_ remains at 37.49 °C in two heating processes, while *T*_endo_ decreases to around 35.20 °C during cooling with a ramp rate of 5 °C/min (Figure 3b). The temperature difference between *T*_exo_ and *T*_endo_ in each cycle is observed in MG320 suspensions, being 3.79 and 3.73 °C in two cycles. In Figure S6, the average temperature difference of MG640 in two cycles increases from 1.35 to 3.56 °C when the ramp rate increases from 1 to 3 °C/min while these values for MG320 suspensions are 1.37 °C at 1 °C/min and 3.48 °C at 3 °C/min.

**Figure 4:**
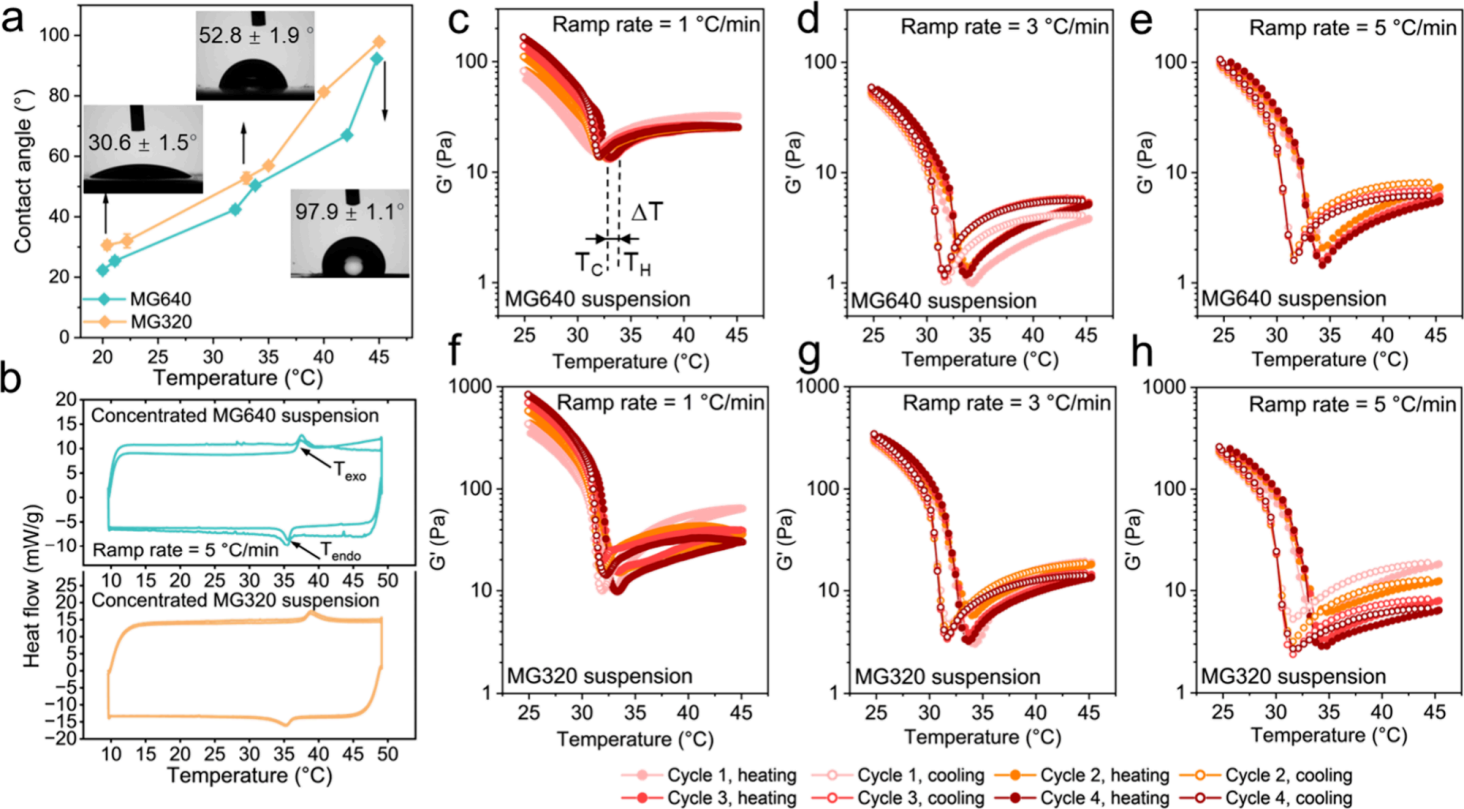
Effect of the temperature ramp rate on concentrated PNIPAm microgel suspensions with different cross-link densities. (a) Contact angles of PNIPAm layers prepared from MG640 and MG320 recipes as a function of temperature. Contact angle images at 20.4, 33, and 45 °C are shown as inserts. (b) DSC thermograms of concentrated MG640 and MG320 suspensions at a ramp rate of 5 °C/min. (c–e) *G*′ of concentrated MG640 suspensions for four cycles of heating and cooling ranging from 25 to 45 °C with ramp rates at (c) 1 °C/min, (d) 3 °C/min, and (e) 5 °C/min. (f–h) *G*′ of concentrated MG320 suspensions for four cycles of heating and cooling ranging from 25 to 45 °C with ramp rates at (f) 1 °C/min, (g) 3 °C/min, and (h) 5 °C/min.

Furthermore, dynamic temperature ramps were performed on concentrated microgel suspensions to study the effect of temperature ramping rate on their rheological properties. To confirm the accuracy of the Peltier heating system of the rheometer, we conducted heating and cooling ramps of DI water on the rheometer at 5 °C/min, the highest rate that will be applied to subsequent experiments, and recorded the temperature shown on the thermometer (Figure S7). The values shown on the rheometer are then used for all subsequent results. Four thermal hysteresis ramps with *G*′ and *G*′′ versus temperature for concentrated PNIPAm microgel suspensions were measured as a function of the ramp rate at a shear rate of 10 rad/s and 1% strain. A butterfly-like shape occurred in all *G*′ and *G*′′ temperature ramp cycling, with additional peaks shown in *G*′′ as a result of the overshoot effect. *T*_H_ is noted as the temperature when *G*′ reaches the lowest value in the heating ramp, while T_C_ indicates the temperature at the lowest *G*′ value in the cooling ramp. Upon heating at 1 °C/min, *G*′ of MG640 microgel suspensions decreases from 139 to 14 Pa at 33.06 °C, due to the decreasing microgel sizes and intermicrogel frictions (Figure 4c). Subsequently, *G*′ slightly increases to 26 Pa at 45 °C owing to the formation of microgel clusters. In a similar manner, *G*′ witnesses this trend in the cooling ramp and reaches the lowest *G*′ at 31.97 °C. Therefore, the average Δ*T* of concentrated MG640 suspensions in four heating and cooling cycles are calculated 0.90, 2.24, and 2.82 °C for ramp rates at 1, 3, and 5 °C/min, respectively (Figure 4c–e). Concentrated MG320 suspensions exhibit similar values of 1.27, 2.24, and 2.92 °C at 1, 3, and 5 °C/min, respectively (Figure 4f–h). With a higher ramp rate, this larger Δ*T* matches with the delayed PNIPAm network collapse that has been confirmed in bulk hydrogels. A similar trend in the heating and cooling ramp, together with Δ*T* at the lowest *G*′ and *G*′′ values, accounts for the butterfly-like curves. The overshoot in *G*′′ happens in both the heating and cooling ramp of MG640 suspension at 1 °C/min, which is primarily attributed to the microgel rearrangement because of the size change (Figure S8). Four heating and cooling ramps also prove the repeatability of microgel shrinkage and the following aggregation. Although an evaporation-avoid setup has been employed, the ramp at 1 °C/min requires more than 160 min to complete, which leads to the elevated *G*′ and *G*′′ concomitantly at each cycle. On the other hand, water evaporation in microgel suspensions barely impacts *G*′ and *G*′′ in ramps at 3 and 5 °C/min. The repeatable temperature responsiveness further confirms the potential in the recycling and reusing PNIPAm microgel suspensions, which broadens their applications as carriers such as drug loading and organic dye removal.^53,54^

### 3.3 Temperature-Dependent Rheological Responses of Diluted PNIPAm Microgel Suspensions

**Figure 5:**
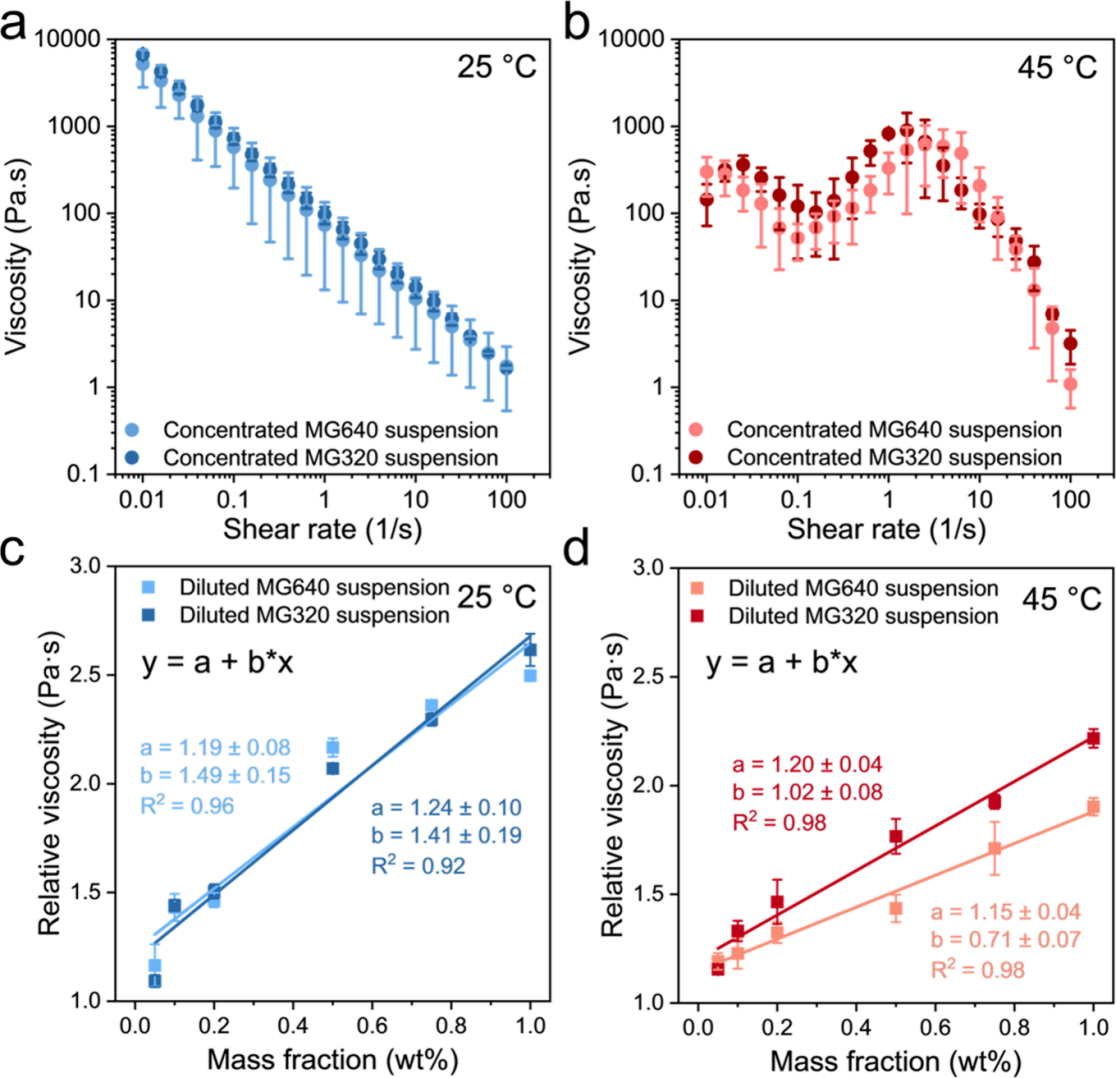
Viscoelastic properties of concentrated and diluted PNIPAm microgel suspensions with different cross-link densities. (a, b) The viscosity of concentrated MG640 and MG320 suspensions at (a) 25 °C and (b) 45 °C, respectively. (c, d) Correlation and linear fitting between the apparent shear viscosity and mass fraction of diluted MG640 and MG320 suspensions at (c) 25 °C and (d) 45 °C, respectively.

The viscosities of concentrated PNIPAm microgel suspensions as a function of shear rate are plotted on a logarithmic scale. Both suspensions display shear-thinning behaviors at 25 °C, thus decreasing viscosity with increasing shear rate (Figure 5a). However, the viscosity values at 45 °C peak in between 1–10 s^–1^, which could presumably be the high shear rate that squeezes microgel clusters out of the geometry (Figure 5b). In contrast to concentrated microgel suspensions, freeze-dried PNIPAm microgels are weighed and rehydrated in DI water with mass fractions of 0.05, 0.1, 0.2, 0.5, 0.75, and 1 wt % to determine the rheological responses of temperature on diluted PNIPAm microgel suspensions. Relative viscosity (*η*_*rel*_) is given by the ratio of the viscosity of a suspension (*η*_*0*_) and the viscosity of DI water (*η*_*s*_), the solvent, at the same temperature. According to the Einstein–Batchelor equation, *η*_*rel*_ is proportional to the particle volume fraction (Φ) in diluted colloidal suspensions.^55^
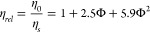


For hard spheres,  and  where *n* is the number of hard particles, *V*_*p*_ is the particle volume, and *r* is the radius of the particle. For highly deformable microgels, the particle volume is no longer the summation of single particles. Senff and Richtering therefore modified the Einstein–Batchelor equation for an experimental fit in viscosity measurements of microgel suspensions.^17^
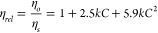
where *k* is the fitting parameter, also known as the transform factor, which transforms the mass fraction of microgels to an effective volume fraction (*Φ*_*eff*_).
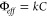


To quantify the relation between *η*_*rel*_ and mass fraction, the *k* value for swelled and deswelled microgels can be derived from the linear fit at temperatures both lower and higher than VPTT. The 40 mm parallel geometry with a gap of 600 μm is employed for all the rheological measurements of diluted microgel suspensions. After reaching the target temperature, the microgel suspensions stabilize for 3 min before conducting subsequent viscosity tests. The solvent trap is filled with DI water, and the immersion ring and chamber are utilized to avoid the evaporation of solvent. The viscosity of DI water and PNIPAm microgel suspensions of different mass fractions are measured between 25 and 45 °C with a shear rate covering 0.01 to 1000 s^–1^ to determine the relative viscosity of diluted microgel suspensions. Linear fitting demonstrates similar slopes of 1.49 and 1.41 for diluted MG640 and MG320 suspensions at 25 °C (Figure 4c). The slope values then move to 0.71 for MG640 and 1.02 for MG320 suspensions at 45 °C, which is attributed to a smaller microgel size for MG640 after dehydration (Figure 4d).

## 4 Conclusion

In conclusion, the effects of cross-link density of PNIPAm microgels and temperature on concentrated and diluted PNIPAm microgel suspensions have been comprehensively studied for the understanding of temperature-responsive microgel suspension systems. A water-in-oil template is applied for the scale-up fabrication of microscale PNIPAm colloids. The prepared microgels of different cross-link densities are then dispersed in DI water and jammed for the concentrated microgel suspensions. Owing to the increasing attraction strength among microgels beyond VPTT, microgels aggregate and form microgel clusters that can be visualized and measured size by DIA. On the other hand, we hypothesize that the microgel–microgel interaction can be neglected in the diluted suspensions, and freeze-dried microgels are weighted and dispersed in water to formulate diluted microgel suspensions of various mass fractions. Rheological responses of concentrated and diluted microgel suspensions are characterized within their LVRs at temperatures below and above VPTT. It is demonstrated that *G*′ and *G*′′ of concentrated microgel suspensions are highly dependent on temperatures and temperature ramp rates. The printability of concentrated microgel suspensions as a function of the syringe and printing bed temperatures is investigated. Butterfly-like curves are observed in the temperature ramps of concentrated microgel suspensions with similar trends in heating and cooling but various Δ*T*. This difference is attributed to the delay in dissolution of polymer chains. Four cycles of heating and cooling further confirm the repeatability of VPT in microgels. In addition, the relative viscosities of diluted microgel suspensions are correlated with mass fractions by linear fitting where the higher slope value indicates larger microgel sizes. We anticipate that our study will provide an in-depth understanding of the temperature-responsive microgel suspensions in both concentrated and diluted manners, enabling finely controllable rheology simply by temperature especially suitable for extrusion-based printing. Potentially, this system can be conveniently combined with biological cells and other functional additives and shows great promise in applications of flexible devices, soft actuators, and biomedical fields.

## Supporting Information Available

FTIR spectra; size distribution of MG640 microgels measured via optical micrographs; size distribution of MG320 microgels measured via optical micrographs; size distribution of MG640 microgel clusters measured via DIA; size distribution of MG320 microgel clusters measured via DIA; DSC thermographs of concentrated MG640 and MG320 suspensions at a ramp rate of 1 °C/min and 3 °C/min; temperature accuracy of the Peltier heating; *G*′′ of concentrated MG640 and MG320 suspensions at a ramp rate of 1, 3, and 5 °C/min (PDF)
Video S1: Cooling down MG640 suspension from 45 to 25 °C (MP4)
Video S2: 3D printing of a three-layer grid structure (MP4)
