# Supplementary material for: 3D Printable Poly(N-isopropylacrylamide) Microgel Suspensions with Temperature-Dependent Rheological Responses
Source: ACS Appl Polym Mater. 2024 Mar 21;6(23):14095–105. doi: 10.1021/acsapm.3c03230 (PMC11650633; doi:10.1021/acsapm.3c03230)
Supplement: Supplementary file 1 — ap3c03230_si_001.pdf [file ap3c03230_si_001.pdf]

Supporting information for

3D printable poly(N-isopropylacrylamide) microgel suspensions with temperature-dependent rheological responses

Zhecun Guan [a] Sai Krishna Katla [b], Vidumin Dahanayake [b], Jinhye Bae [a,c,d*]

a. Department of NanoEngineering, University of California San Diego, La Jolla, CA 92093, USA.

b. Anton Paar USA, Inc., Ashland, VA 23005, USA

c. Chemical Engineering Program, University of California San Diego, La Jolla, CA 92093, USA.

d. Materials Science and Engineering Program, University of California San Diego, La Jolla, CA 92093, USA.

Corresponding author:

Dr. Jinhye Bae, Email: j3bae@ucsd.edu

KEYWORDS

Microgels; stimuli-responsive hydrogels; rheological response; temperature-responsiveness; 3D printing.

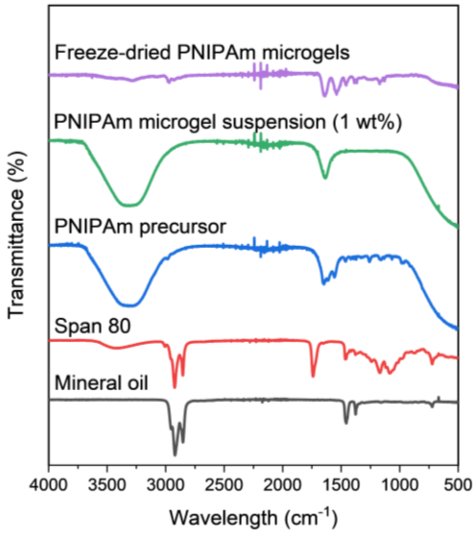

Figure S1. FTIR spectra of freeze-dried PNIPAm microgels, PNIPAm microgel suspension at 1 wt%, PNIPAm precursor, Span 80, and mineral oil.

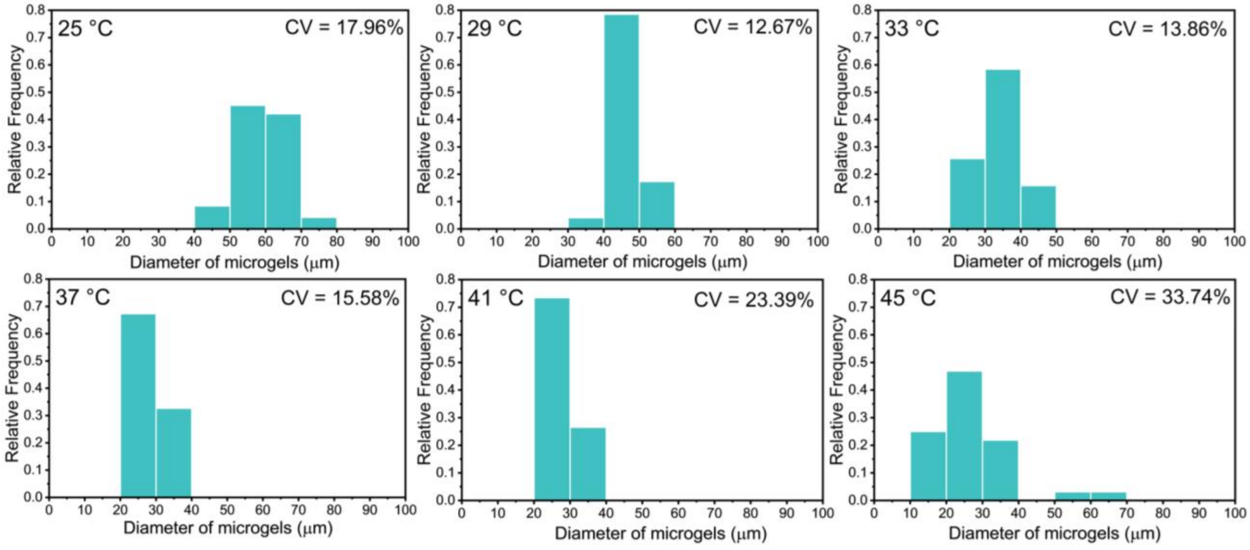

Figure S2. Size distribution of MG640 microgels at different temperatures measured via optical micrographs.

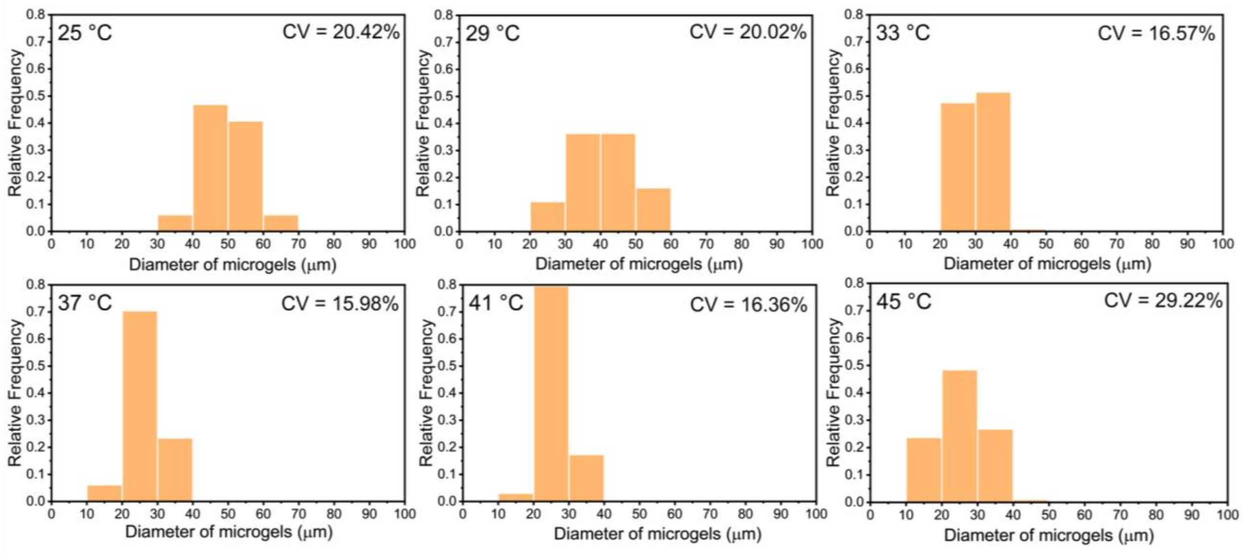

Figure S3. Size distribution of MG320 microgels at different temperatures measured via optical micrographs.

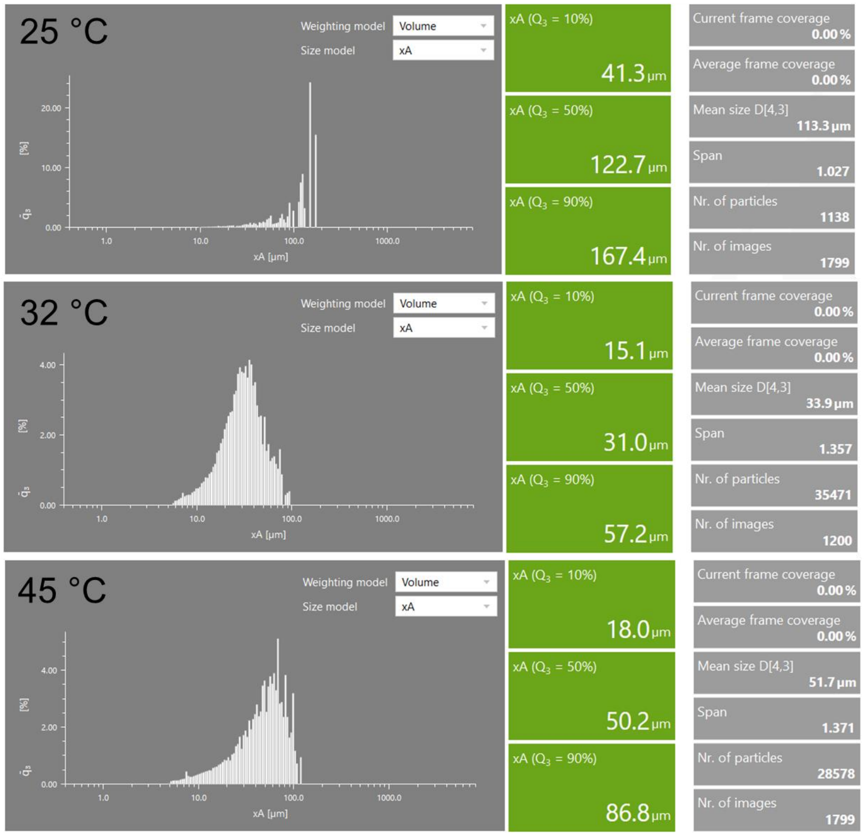

Figure S4. Size distribution of MG640 microgel clusters in one measurement at different temperatures measured at 25, 32, and 45 °C by DIA in Kalliope software. Microgel cluster distribution shown in Figure 1g contains three measurements at the same condition.

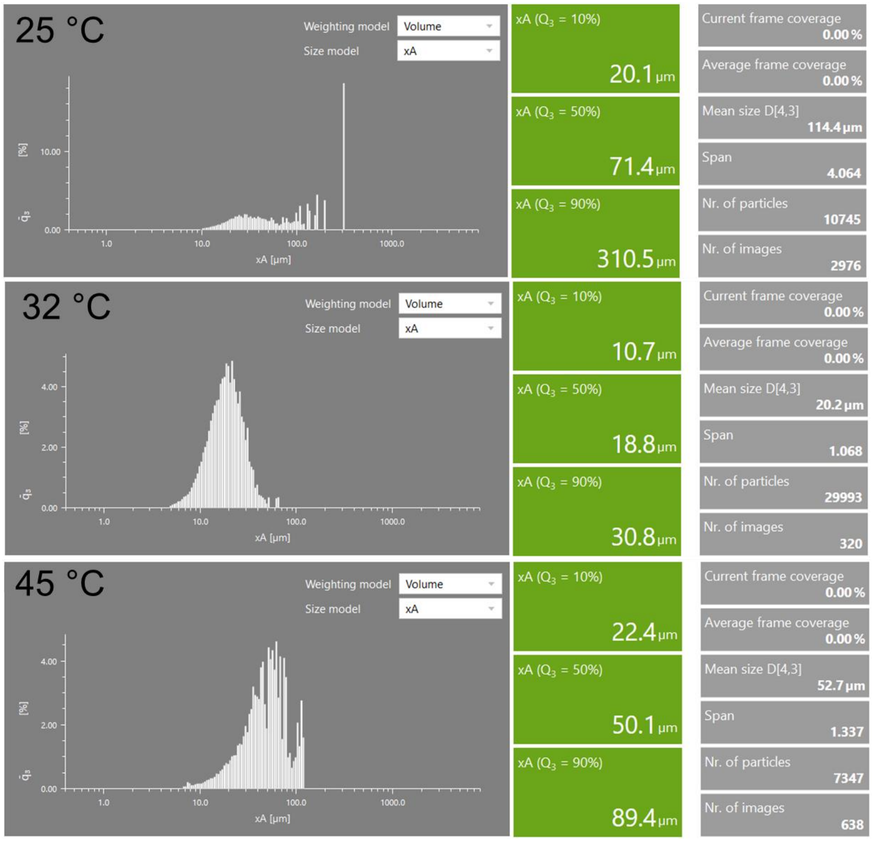

Figure S5. Size distribution of MG320 microgel clusters at different temperatures measured at 25, 32, and 45 °C by DIA.

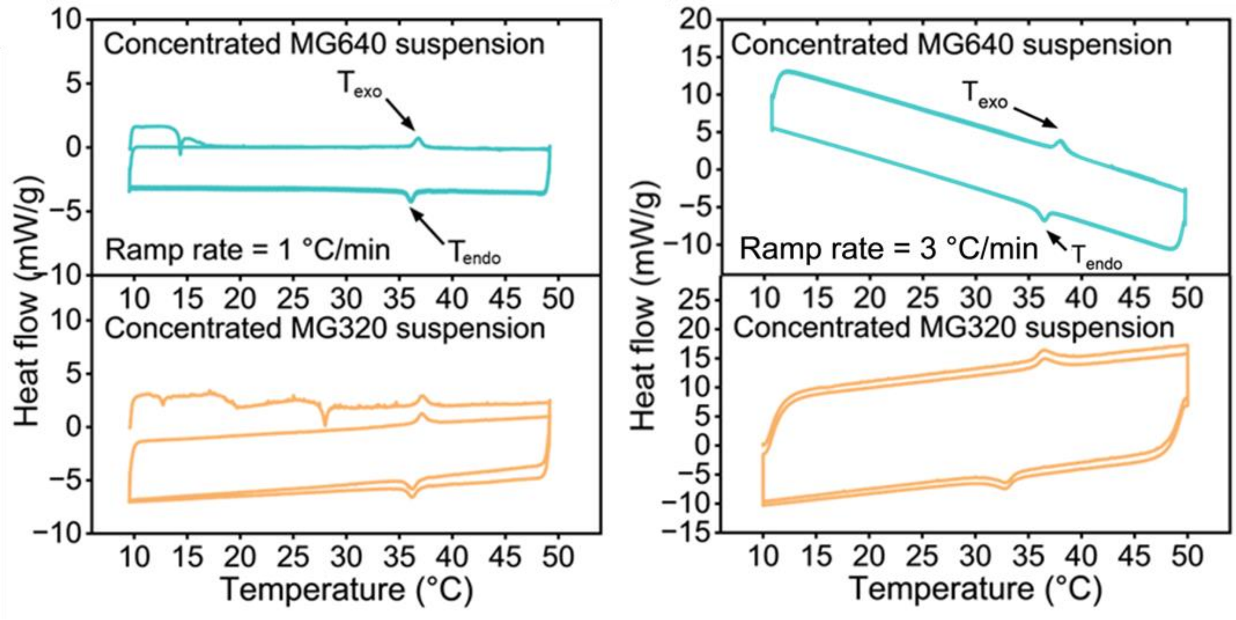

Figure S6. DSC thermographs of concentrated MG640 and MG320 suspensions at a ramp rate of (a) 1 °C/min and (b) 3 °C/min for two heating and cooling cycles between 10 °C and 50 °C. The fluctuations in the first heating cycle for concentrated MG640 and MG320 suspensions with a ramp rate of 1 °C/min are primarily attributed to the differences in the heat capacity of the sample and reference sample and a relatively low resolution of ramp rate.[1]

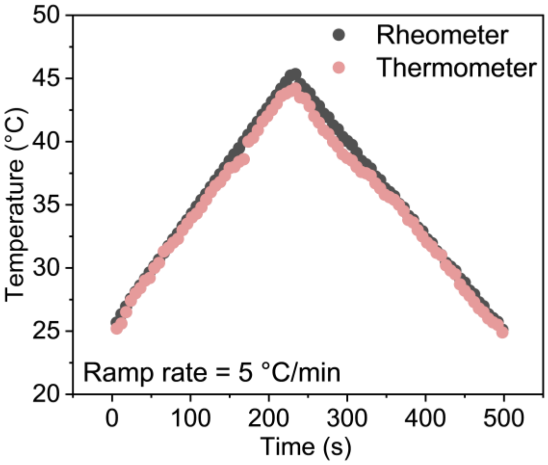

Figure S7. Temperature accuracy of the Peltier heating value shown on the rheometer and measured by thermometer at a ramp rate of 5 °C/min.

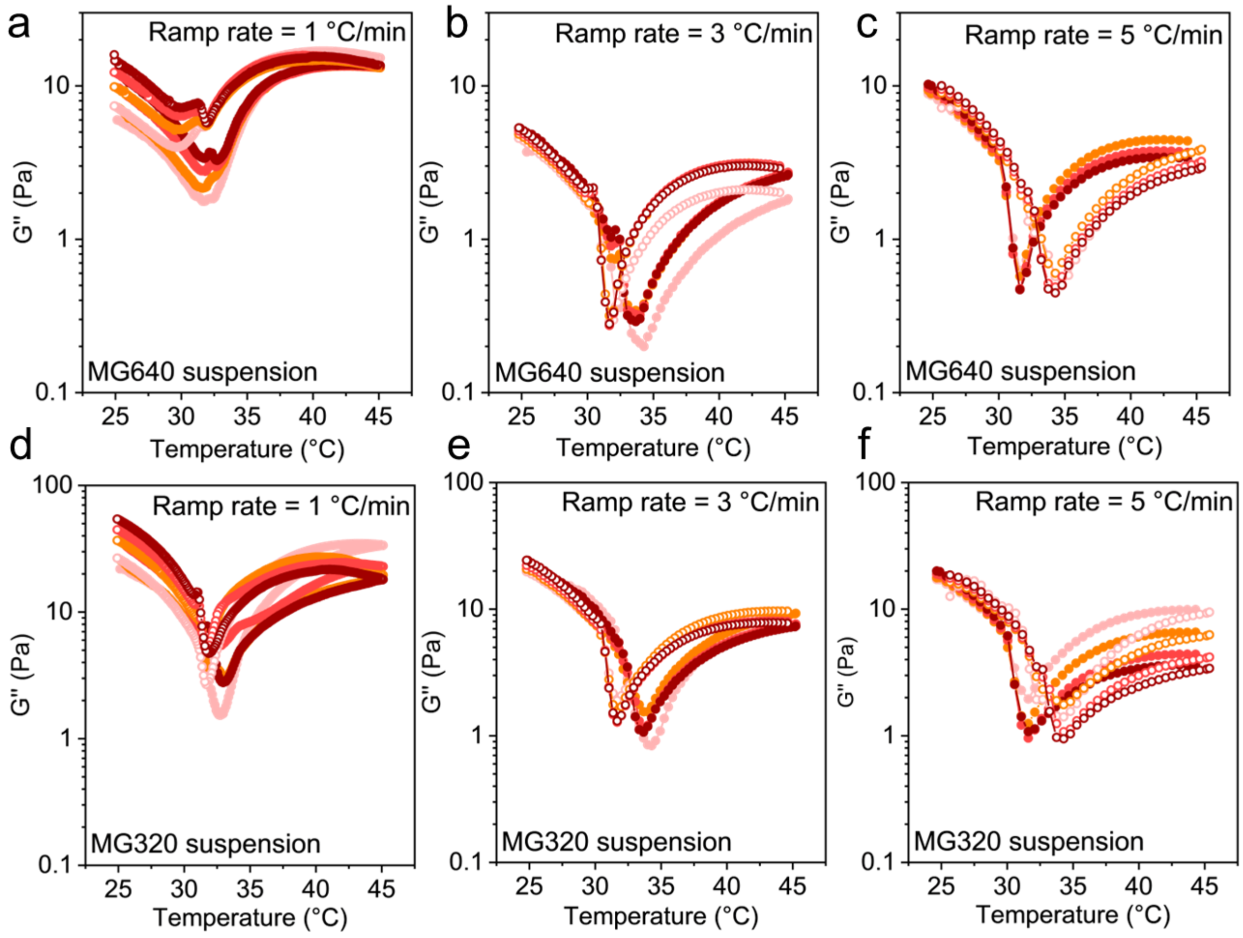

Figure S8. G” of concentrated MG640 (a, b, c) and MG320 (d, e, f) microgel suspensions for four heating and cooling cycles from 25 °C to 45 °C at a ramp rate of 1°C /min, 3 °C /min, and 5 °C/min, respectively.

Supporting videos

Video S1. Cooling down MG640 suspension from 45 °C to 25 °C.

Video S2. 3D printing of a three-layer grid structure.

References

1. Mohamed, R.; Abd Razak, R.; Abdullah, M. M. A. B.; Shuib, R. K.; Mortar, N. A. M.; Zailani, W. W. A. In *Investigation of heat released during geopolymerization with fly ash based geopolymer*, IOP Conference Series: Materials Science and Engineering, IOP Publishing: 2019; p 012093.
